# Perceived Parenting and Adolescent Cyber-Bullying: Examining the Intervening Role of Autonomy and Relatedness Need Satisfaction, Empathic Concern and Recognition of Humanness

**DOI:** 10.1007/s10826-016-0401-1

**Published:** 2016-03-09

**Authors:** Kyriaki Fousiani, Panagiota Dimitropoulou, Michalis P. Michaelides, Stijn Van Petegem

**Affiliations:** Faculty of Behavioral and Social Sciences, University of Groningen, Groningen, The Netherlands; Department of Primary Education, University of Ioannina, Ioannina, Greece; Department of Psychology, University of Cyprus, Nicosia, Cyprus; Family and Development Research Center (FADO), Institute of Psychology, University of Lausanne, Lausanne, Switzerland

**Keywords:** Cyber-bullying offending, Parental autonomy support, Parental psychological control, Autonomy, Relatedness, Empathic concern, Humanness

## Abstract

Due to the progress in information technology, cyber-bullying is becoming one of the most common forms of interpersonal harm, especially among teenagers. The present study (*N* = 548) aimed to investigate the relation between perceived parenting style (in terms of autonomy support and psychological control) and cyber-bullying in adolescence. Thereby, the study tested for the intervening role of adolescent need satisfaction (i.e., autonomy and relatedness), empathic concern towards others, and adolescents’ recognition of full humanness to cyber-bullying offenders and victims. Findings revealed both a direct and an indirect relation between parenting and cyber-bullying. More specifically, parental psychological control directly predicted cyber-bullying, whereas parental autonomy support related to less cyber-bullying indirectly, as it was associated with the satisfaction of adolescents’ need for autonomy, which predicted more empathic concern towards others, which in turn differentially related to recognition of humanness to victims and bullies. The discussion focuses on the implications of the current findings.

## Introduction

Cyber-bulling constitutes a common problem in adolescence, as a meaningful proportion of teenagers is involved in such experiences. Cyber-bullying can be defined as the aggressive and intentionally repeated act conducted by an individual or a group of individuals using technology for electronic contact against a victim (Smith et al. 2008). It includes every experience with any form of online harassment and may impact its victims on various levels (e.g., psychological, emotional, social, etc.; Hinduja and Patchin 2009).

Although cyber-bullying and traditional bullying are taking place on a different and unique venue, there generally is a consensus among researchers that they function in quite similar ways, including the aggressiveness characterizing online and offline bullying, and the imbalance of physical, social, relational, or psychological power between bully and victim. Further, the behavior is repeatedly displayed, with the intention to harm another individual (Olweus 2013). In addition, research has shown that individuals involved in traditional bullying also often get involved in cyber-harassment (Hemphill et al. 2012). These similarities have led researchers to suggest that cyber-bullying and traditional bullying are closely related, pointing out that electronic media is another medium through which individuals who already aggress offline, can now aggress online as well (Werner et al. 2010).

However, cyberbullying is characterized by certain unique features as well (Dooley et al. 2009), such as the possible *anonymity* of the offender and the *distancing* effect that technological devices ensure, rendering cyberbullies more unaware about the consequences of their behaviors—a fact that, in turn, may reduce potential empathy towards their victims. This may explain why cyberbullies may treat their victims in an even harsher way than typical face-to-face bullies. Moreover, given how fast and how widespread such harmful material against cyberbullying victims can be shared through mobile phones and through the Internet, the *group effect* constitutes a further aspect of cyberbullying that may render this form of bullying as more distressful and damaging for the victim (see Dooley et al. 2009, for a review). For the above reasons, we viewed cyberbullying as a more crucial form of bullying in the modern society, and thus, we focused on this form rather than traditional bullying.

Numerous studies have revealed the important role of the parenting context for understanding the development of bullying and victimization. For instance, bullies often describe their family as authoritarian and less organized, whereas victims rather view their parents as highly permissive (Baldry and Farrington 2000). Similarly, cyber-bullying offenders often report experiencing limited parental monitoring, stronger parental discipline and a weaker emotional bond with their parents, as compared to those not engaging in cyber-bullying (Wang et al. 2009). Herein, we focused specifically on two parenting dimensions that gained relatively less empirical attention, that is, parental autonomy support (AS) and psychological control (PC).

Parental AS is defined as the promotion of youngsters’ volitional functioning, in which case parents are empathic towards the adolescent’s perspective, provide choice whenever possible, and provide a meaningful rationale when choice is limited (Grolnick 2003; Soenens et al. 2007). By doing so, parents encourage their children to make self-endorsed decisions and choices that reflect their internalized values, preferences and interests (e.g., Fousiani et al. 2014). Parental PC, on the other hand, involves the use of manipulative and coercive tactics such as love withdrawal, instilling anxiety, guilt induction and invalidation of the child’s perspective (Barber and Harmon 2002; Soenens and Vansteenkiste 2010). Several studies among adolescents have shown that parental AS relates to higher psychosocial adjustment, whereas PC is associated with maladjustment and psychopathology (for a review, see Soenens and Vansteenkiste 2010). In addition, children of highly psychologically controlling parents also would engage more often in manipulative and relationally aggressive behaviors such as gossiping, damaging other people’s social reputation and threatening to end a friendship (e.g., Soenens et al. 2008), behaviors that are common to cyber-bullying offending. In a similar vein, studies that confirmed the associations between (autonomy-supportive versus controlling) teaching practices and bullying and violence also offer indirect evidence for the relevance of parental AS (vs. PC) for understanding adolescent cyber-bullying (Hein et al. 2015; Roth et al. 2011).

According to Self-Determination Theory (Ryan and Deci 2000; Soenens and Vansteenkiste 2010), the beneficial (vs. detrimental) correlates of parental AS (vs. PC) can be explained by adolescents’ perceived satisfaction (vs. frustration) of their basic psychological needs for autonomy, relatedness and competence, which would be essential for an entity’s growth and healthy development (Ryan 1995). Thereby, the need for *autonomy* implies that people have a natural desire to experience their behavior as volitional and personally relevant (Deci and Ryan 1985). *Relatedness* implies that people want to care for others and to feel cared by them (Ryan 1995). Finally, *competence* refers to one’s desire to feel effective and skillful in the activities one undertakes (Ryan 1995). A growing body of research points to the explanatory role of adolescents’ need satisfaction in the relation between perceived parental AS and PC, and adolescents’ adjustment. The more parents are perceived as autonomy-supportive, the more children feel self-determined and autonomous, experience positive relations, and feel more effective in their actions, which predicts higher well-being, whereas the opposite is true for PC (e.g., Ahmad et al. 2013; Inguglia et al. 2015).

Although no research to date explicitly tested the role of need satisfaction in cyber-bullying/victimization matters, there is evidence that need satisfaction, and the satisfaction of autonomy and relatedness in particular, relates to antisocial and aggressive behavior. Weinstein et al. (2011), for instance, found that low autonomy is associated with the enjoyment of hostile humor, whereas Van Petegem et al. (2015) provided evidence for the explanatory role of autonomy frustration in the relation between parental PC and externalizing symptoms. As far as relatedness is concerned, Park et al. (2011) found that relatedness was associated with more altruism, whereas Miklikowska et al. (2011) found support for the longitudinal relation between perceived need-supportive parenting and empathy among adolescents. However, contrary to need for autonomy and relatedness, there is less evidence that general competence satisfaction would relate to either aggression or bullying (for exceptions, see the work on specific competences, such as social competence, Irshad and Atta 2013, and moral competence, Gasser and Keller 2009).

Another major element of bullying behavior that may be relevant is the power imbalance between the parties involved (Olweus 2013). Power imbalance often involves the perceived superiority of harm-doers and inferiority of those being harmed. In that respect, perceived superiority versus inferiority also may pertain to recognition of human essence to each party involved in bullying, where human essence refers to the elements that distinguish people from animals or objects rendering humans superior (see e.g., Haslam 2006). Literature indicates that oppression or marginalization of humans may be rooted in the human-animal or even human-object division, where marginalized or oppressed groups are seen as less-than-human (Livingstone-Smith 2011). Among children, there is limited research evidence linking anti-social behavior and bullying with such dehumanizing practices against victims (Costello and Hodson 2012; van Noorden et al. 2014).

According to the existing literature, harm-doers would be enabled to commit horrible acts through a denial of the others’ humanness, a core dimension of the well-known “moral disengagement” phenomenon. Moral disengagement is the process by which people convince themselves that detrimental conduct directed toward individuals is morally acceptable by converting harmful acts to moral ones through linkage to worthy purposes (Bandura 1986; Obermann 2011). In social psychology, dehumanization theory (see Haslam 2006) has been used frequently for explaining aggressive and violent behaviors against individuals or groups. Two types of human characteristics can be distinguished to individuals: (a) *uniquely human* (*UH*) characteristics (e.g., civility, morality, rationality) are the ones distinguishing humans from animals and they involve high-order cognition. Denial of this kind of traits to individuals is called animalistic dehumanization (Haslam 2006) and it involves categorization of others as inferior beings, hence justifying aggressive or violent behaviors against others; (b) *human nature* (*HN*) characteristics (e.g., emotional responsiveness, cognitive openness) distinguish people from machines or automata. Denial of these characteristics is called mechanistic dehumanization (Haslam 2006) and it involves viewing the others as emotionally cold, close-minded and passive just like objects and it serves in treating them with psychological distance or indifference.

Interestingly, it seems that denial of UH or HN characteristics to the others could be related to experiences of autonomy and relatedness frustration. We are aware of only one study to date that has revealed the explanatory role of denial of HN traits to others in the relation between autonomy frustration and interpersonal violence and aggression (Moller and Deci 2010). However, denial of humanness to others also is linked to decreased empathic skills of harm-doers, whereas empathy is considered as a requirement for overcoming dehumanization (Halpern and Weinstein 2004). According to the literature, empathy encompasses two dimensions: (a) an affective dimension, often referred to as *empathic concern,* which represents the ability to experience another’s emotions; and (b) a cognitive dimension, often referred to as *perspective taking,* which reflects the ability to understand another person’s emotional state (Gini 2006). Several studies, measuring both kinds of empathy, revealed the significance of affective over cognitive empathy in bullying/victimization contexts (Stavrinides et al. 2010). Therefore, in this study we focused on adolescents’ capacity for empathic concern. Moreover, as for the association with recognition of humanness to the others, Capozza et al. (2013) and Čehajić et al. (2009) revealed that, when people do recognize uniquely human emotions to victims, they experience more empathy towards them.

The present study tests for the relation between perceived parental AS and PC and adolescents’ involvement in cyber-bullying. Thereby, we tested for the intervening role of adolescents’ need satisfaction (in terms of autonomy and relatedness), empathic concern, and their inclination to recognize humanness to both bullies and victims. We hypothesized that perceived parental AS (as opposed to PC) would relate to less cyber-bullying, both directly and indirectly through need satisfaction and empathic concern. In addition, it was expected high empathy would relate to the recognition of full humanness to victims and to decreased recognition of humanness to bullies. High recognition of humanness to victims and low recognition to bullies, in turn, would be associated with lower levels of cyber-bullying. Furthermore, it was hypothesized that UH characteristics would play a more significant role in cyber-bullying as compared to HN characteristics, as UH characteristics especially would be related to moral disengagement (Haslam 2006).

## Method

### Participants

The sample was composed of 548 high school (Grades 10–11) students (48.2 % male) from 11 schools in Nicosia, the capital of Cyprus. 61.2 % were from the first class of high school, 38.8 % from the second class and 0.2 % from the third class. Respondents came from intact (77.4 %), divorced (16.1 %) or single-parent (6.5 %) families. 95.4 % of the students were connected on a social network (e.g., Facebook, Twitter). Participation in the study was voluntary and anonymity was guaranteed. Participants’ socio-economic status (SES) was not explicitly measured, but given that the sample consisted of adolescents from various districts of the capital of Cyprus, it is likely that the sample strongly varied in terms of SES.

### Procedure

Questionnaires were administered during a regular class period at school. The study procedure was in line with the criteria set by Cyprus authorities and with the regulations about ethical issues. Specifically, a permission was obtained by the Cyprus Ministry of Education and Culture as well as the Pedagogical Institute of Cyprus, which is responsible for research affairs in schools in Cyprus.

### Measures

Greek versions of the parenting scales already have been used successfully in Greek samples in prior research (Fousiani et al. 2014). To the best of our knowledge, the other scales have not been used in a Greek-speaking sample before, and thus were translated into Greek by the authors through the same procedure as with the parenting scales, that is, a translation-back translation procedure.

#### Perceived Parental Autonomy Support (AS)

Parental AS was measured through the Autonomy Support subscale of the Perceptions of Parenting Scale (POPS; Grolnick et al. 1991). This questionnaire consists of seven items (e.g., “whenever possible, my mother allows me to choose what to do.”). Items were rated only for mothers. Participants answered on a 7-point Likert-type scale, ranging from 1 (“not at all true”) to 7 (“absolutely true”). Extensive validity information of the scale is provided by Soenens et al. (2007). The psychometric characteristics of the Greek version of the scale had been found to be satisfactory as well (Fousiani et al. 2014). In the present study, Cronbach’s α was .84.

#### Perceived Parental Psychological Control (PC)

Respondents completed the 8-item Psychological Control Scale-Youth Self Report (Barber 1996; e.g., “my mother is always trying to change how I feel or think about things”) for the assessment of maternal PC. Participants answered on a 7-point Likert-type scale, ranging from 1 (“not at all true”) to 7 (“absolutely true”). In the present study, Cronbach’s α was .79.

#### Basic Psychological Need Satisfaction

To assess basic psychological need satisfaction, we used two subscales from the Basic Psychological Need Satisfaction and Need Frustration Scale (BPNSNFS; Chen et al. 2015). Autonomy need satisfaction was measured through eight items, of which four items assessing autonomy satisfaction (i.e., “I feel a sense of choice and freedom in the things I undertake”) and four items assessing autonomy frustration (i.e., “I feel forced to do many things I wouldn’t choose to do”). Likewise, relatedness satisfaction was assessed through eight items as well, of which four items measuring relatedness satisfaction (i.e., “I feel that the people I care about also care about me”) and four items measuring relatedness frustration (i.e., “I feel that people who are important to me are cold and distant towards me”). Participants responded on a scale from 1 (“not at all true”) to 7 (“absolutely true”). Extensive validation information about the cross-cultural applicability of the BPNSNFS is provided by Chen et al. (2015). Cronbach’s α were .75 for autonomy satisfaction, .65 for autonomy frustration, .80 for relatedness satisfaction and .76 for relatedness frustration.

#### Recognition of Humanness to Bullies and Victims

Participants rated four items for the assessment of recognition of uniquely human (UH) traits of bullies and the same four items for recognition of UH traits of victims; three items were administered for the assessment of recognition of human nature (HN) traits of victims and of bullies (Bastian and Haslam 2010; Haslam 2006). Both scales were administered separately for bullies and victims. Examples items are “bullies/victims are rational and logical” (recognition of UH) and “bullies/victims are emotional, responsive and warm in their interpersonal relations” (recognition of HN). Responses were given from 1 “strongly disagree” to 7 “strongly agree”. One item was dropped for recognition of HN traits of victims after scale analyses. Cronbach’s *α* were .81 and .80 for bullies’ UH and HN trait scales respectively, and .83 and .77 for victims’ UH and HN traits scales respectively.

#### Cyber-Bullying Offending

The well-validated 5-item cyber-bullying scale (Hinduja and Patchin 2009) was used for the assessment of cyber-bullying offending. Questions assessed the frequency of cyber-bullying offending behaviors (e.g., In the last 30 days,… “…I have posted something online about another person to make others laugh”, “…I have sent someone a computer text message to make them angry or to make fun with them”). Responses were rated on a 7-point scale (from 1 “never”, to 7 “very often”), with higher scores indicating more frequent offending behaviors. Cronbach’s α was .86.

#### Empathic Concern

The 7-item Empathic concern (EC) subscale of the Empathy Scale (Davis 1983) was used to assess “other-oriented” feelings of sympathy and concern for unfortunate others. Questions were rated on a 7-point Likert scale ranging from 1 (“does not describe me at all”) to 7 (“describes me very well”). This often-used questionnaire has been found to be valid and reliable in previous research (e.g., Miklikowska et al. 2011). In the present study, Cronbachs’ α of the scale was .70.

### Data Analyses

A full latent structural equation model was employed to investigate the hypotheses of the study. The covariance matrix of all items was analyzed using maximum likelihood estimation in AMOS 20. Prior to examining the structural models, a series of measurement models were analysed for the formulation of the latent variables. Perceived parental Autonomy Support (AS) and Psychological Control (PC) served as exogenous, correlated, latent variables; the former was modeled on five reflective indicators from the AS-subscale of the POPS and the latter was modelled on eight reflective indicators of the Psychological Control Scale-Youth Self Report. Autonomy and Relatedness needs were modelled as correlated, endogenous, second-order variables. A Satisfaction and a Frustration first-order factor loaded on each of the two basic needs variables. Each Satisfaction and each Frustration variable comprised of four items from the *BPNSNFS*. Empathic Concern was an endogenous variable consisting of seven empathic concern items from the Empathy scale. Human Nature and Human Uniqueness of bullies were allowed to correlate and consisted of three and four items, respectively. Human nature and Human uniqueness of victims were allowed to correlate and consisted of two and four items respectively. Cyber-Bullying, an endogenous variable, consisted of five items obtained from the Cyber-Bullying Offending Scale.

A sequence of three fully latent structural equation models were then tested: Model 1 included AS and PC as exogenous predictors for Autonomy and Relatedness factors. All four factors were used as predictors of Cyber-Bullying. For Model 2, the Empathic Concern factor was added as an intervening variable between Autonomy and Relatedness factors and Cyber-Bullying. Finally, Model 3 introduced the four humanness factors as intervening variables between Empathic Concern and Cyber-bullying. At each stage, model fit was assessed and non-significant paths were deleted before moving to the subsequent model.

Overall model fit was evaluated with the χ^2^-statistic. However, because this statistic is sensitive to sample size and may overestimate the lack of model fit, the following goodness-of-fit indices were also examined: the Comparative Fit Index (CFI), the Standardized Root Mean square Residual (SRMR), and the Root Mean Square Error of Approximation (RMSEA), along with a 90 % confidence interval. Values .90 or higher for CFI, less than .08 for SRMR and less than .05 for RMSEA were taken as evidence of adequate fit between a hypothesized model and the data.

## Results

Means, standard deviations and correlation coefficients among all variables in the study appear in Table 1. The participants reported relatively high average scores on autonomy support, autonomy and relatedness satisfaction, and empathic concern. Average scores for psychological control, relatedness frustration, human uniqueness and nature of bullies and cyber-bullying were rather low. Correlation coefficients generally were in the expected direction. Cyber-bullying in particular related positively to psychological control, autonomy and relatedness frustration, and recognition of human characteristics to bullies; it related negatively to relatedness satisfaction, empathic concern and recognition of human uniqueness to victims.

**Table 1:** Descriptive statistics for the variables in the study

	# items	Mean	SD	2	3	4	5	6	7	8	9	10	11	12
1. AS	5	5.45	1.15	−.47**	.48**	−.20**	.39**	−.25**	.21**	−.09*	.09*	−.09*	.04	−.05
2. PC	8	2.43	1.08		−.28**	.38**	−.31**	.39**	−.11*	.21**	−.02	.18**	.06	.21**
3. Autonomy satisfaction	4	5.42	1.15			−.19**	.44**	−.30**	.21**	−.12**	.08	−.11*	.08	−.01
4. Autonomy frustration	4	3.82	1.28				−.08	.34**	−.02	.12**	.00	.15**	.06	.12**
5. Relatedness satisfaction	4	5.82	1.05					−.52**	.19**	−.15**	.08	−.14**	.10*	−.11**
6. Relatedness frustration	4	2.31	1.20						−.03	.19**	−.02	.17**	.06	.18**
7. Empathy Concern	7	5.45	.90							−.30**	.15**	−.24**	.09*	−.28**
8. UH Bullies	4	1.93	1.20								.02	.72**	.05	.31**
9. UH Victims	4	4.07	1.52									.03	.61**	−.15**
10. HN Bullies	3	2.00	1.36										.06	.24**
11. HN Victims	2	4.07	1.70											−.07
12. Cyber-bullying	5	1.74	1.12											

*AS* autonomy support, *PC* psychological control, *UN* uniquely human characteristics, *HN* human nature traits

* *p* < .05; ** *p* < .01

**Fig. 1:**
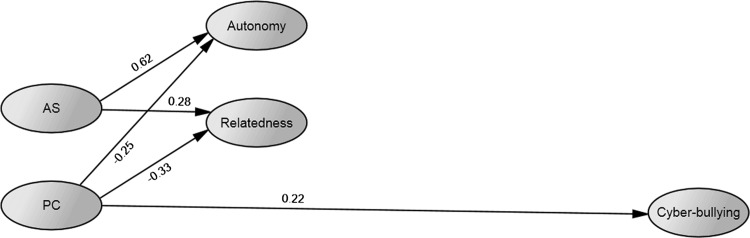
Model 1 after trimming. *Note* All variables are latent, indicators and covariances between AS and PC and between Autonomy and Relatedness are not depicted on the figure. *Numbers* represent standardized estimates of regression paths. *AS* autonomy support, *PC* psychological control

The first model was specified with AS and PC as predictors of Cyber-bullying. Autonomy and Relatedness were also included as intervening variables between the parenting variables and Cyber-bullying. After removing the non-significant paths, the fit of the model was acceptable ($\chi_{(510)}^{2}$ = 957.021, *p* < .001, *CFI* = .929, *RMSEA* = .040, 90 % CI [.036–044], *SRMR* = .055), and is depicted in Fig. 1. AS was not significantly related to cyber-bullying, but significantly related to more autonomy satisfaction and relatedness satisfaction, with a particularly strong relation with autonomy. PC had a small to moderate negative association with the needs variables, and a small positive relation with cyber-bullying. Autonomy and relatedness were not significantly related to cyber-bullying.

**Fig. 2:**
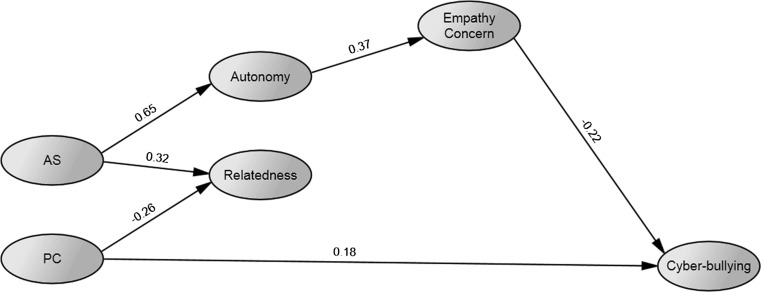
Model 2 after trimming. *Note* All variables are latent, indicators and covariances between AS and PC and between Autonomy and Relatedness not depicted on the figure. *Numbers* represent standardized estimates of regression paths. *AS* autonomy support, *PC* psychological control

Next, empathic concern was entered in the analysis. Paths from autonomy and relatedness were specified on empathic concern, and a path from the latter on cyber-bullying. The analysis resulted in a non-positive definite matrix; an inadmissible correlation estimate was found between the disturbances of the first-order factors of autonomy satisfaction and relatedness satisfaction. In a test of critical ratios for differences in Model 1, the two disturbances were found to be not significantly different. Constraining them to be equal led to an admissible solution. Non-significant path estimates were removed and the resulting model had an acceptable fit: $\chi_{{\left( {759} \right)}}^{2}$ = 1435.449, *p* < .001, *CFI* = .906, *RMSEA* = .040, 90 % CI [.037–.044], *SRMR* = .062. In this model (Fig. 2), AS had significant path coefficients on the needs variables. The coefficient from PC on autonomy was non-significant and those on relatedness and cyber-bullying were significant but slightly lower in magnitude compared to Model 1. Empathic concern was associated positively with Autonomy, but not with Relatedness. Empathic concern, in turn, predicted less Cyber-bullying.

**Fig. 3:**
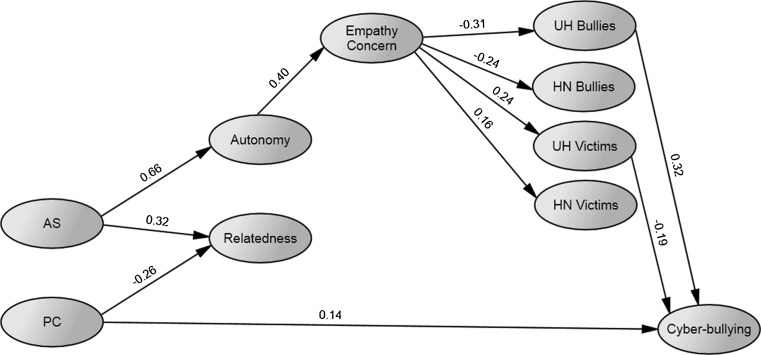
Model 3 after trimming. *Note* All variables are latent, indicators and covariances between AS and PC, between Autonomy and Relatedness, between the humanness variables for bullies, and between the humanness variables for victims not depicted on the figure. *Numbers* represent standardized estimates of regression paths. *AS* autonomy support, *PC* psychological control, *UN* uniquely human characteristics, *HN* human nature traits

Subsequently, four latent factors representing the humanness variables were introduced as well. Paths from empathic concern on the humanness variables, and from the humanness variables on cyber-bullying were added. After removing non-significant paths, the final Model 3 (Fig. 3) had the following fit indices: $\chi_{{\left( {1350} \right)}}^{2}$ = 2408.293, *p* < .001, *CFI* = .901, *RMSEA* = .038, 90 % CI [.035–.040], *SRMR* = .065. Compared to Model 2, the regression estimates from the parenting variables to the needs factors and on Empathic concern were similar. The direct effect from empathic concern on cyber-bullying was no longer significant, though the former had significant path coefficients on all four humanness variables. Specifically, empathic concern related to less humanness attributed to bullies and more humanness attributed to victims. Relations with human uniqueness appeared to be stronger than with the human nature factors for both bullies and victims. Further, cyber-bullying related positively to human uniqueness of bullies and negatively to human uniqueness of victims.

## Discussion

Currently, cyber-bullying constitutes one of the most common forms of interpersonal harm among teenagers with increasingly serious social and personal ramifications (Smith et al. 2008). However, factors contributing to the manifestation of such aggressive behaviors among adolescents on cyberspace still remain largely unexplored. The major focus of this study lies in the holistic consideration of this topic taking into account both parental as well as adolescent factors, trying to test both their direct and indirect relations with cyber-bullying. More specifically, this study had two major goals, that is (a) exploring the direct relations between perceived parental autonomy support and psychological control, and cyber-bullying among adolescents, and (b) investigating the possible intervening role of a number of variables, namely, adolescents’ autonomy and relatedness need satisfaction, empathic concern towards others, and recognition of humanness to both bullies and victims.

As for the first goal of the study, past research has systematically indicated the importance of autonomy support in youngsters’ pro-social stance (e.g., Miklikowska et al. 2011; Roth et al. 2011); the current findings manifested a positive direct relation between perceived parental psychological control and cyber-bullying. In addition, the findings obtained in this study reveal an indirect relation between perceived parental autonomy support and cyber-bullying. Specifically, autonomy support related positively to the satisfaction of adolescents’ need for relatedness and autonomy. In turn, when the need for autonomy was satisfied adolescents reported higher capacities in responding to the others’ emotions, which in turn related to their capacity to recognize civilized and moral individuals and thus delegitimize harm-doers (i.e., bullies).

Thereby, empathic concern differentially related to adolescents’ recognitions of humanness to bullies and victims. Specifically, it related positively to recognition of both human uniqueness and human nature traits to the victims (i.e., humanization of the victims) and negatively to the recognition of both types of humanness to bullies (i.e., dehumanization of bullies). Such findings are in accordance with Gini’s (2006) study which showed that bullies display significant deficiencies with respect to moral sentiments and caring, and higher levels of moral disengagement. In a similar vein, recent research has revealed a positive association between bullying behavior among adolescents and callous-unemotional traits, namely, lack of guilt, lack of empathy, and uncaring (Viding et al. 2009). Interestingly, although empathic concern predicted both human uniqueness and human nature characteristics, only human uniqueness characteristics were related to cyber-bullying behavior. These results are consistent with the literature, as denial of human uniqueness traits is related to disgust to others, humiliating treatment, moral disengagement and delegitimization of others, whereas denial of human nature traits is mostly associated with indifference to others and psychological distance (for a review see Haslam 2006; Haslam and Loughnan 2014). In line with our findings, van Noorden et al.’s (2014) study has demonstrated the importance of animalistic dehumanization (i.e., denial of human uniqueness)—but not mechanistic dehumanization (i.e., denial of human nature)—in bullying situations.

Contrary to our expectations, the need for relatedness did not relate to cyber-bullying, neither directly nor indirectly. Although bullying at a first glance would refer to relatedness issues, as it involves violence and aggression between individuals or groups, the findings of the current study seem to suggest that the frustration of need for autonomy especially has a stronger association with cyber-bullying. A number of other studies indirectly support our findings that especially autonomy frustration is important for understanding anti-social behaviors. For instance, when people feel that their autonomy has been thwarted, they often respond in a more anti-social manner involving increased anger and aggression (Neighbors et al. 2002), social dominance and racial prejudice (Duriez et al. 2007). On the contrary, when the need for autonomy is satisfied more pro-social attitudes and behaviors (Gagne 2003) and less moral disengagement (Mask et al. 2005) are displayed. Possibly, relatedness frustration may be more closely linked to more internalizing types of problems. Indeed, previous studies on loneliness (which may be indicative of relatedness frustration) especially documented associations with internalizing problems, including anxiety, depression, and suicidal ideation (Ernst and Cacioppo 1999; Heinrich and Gullone 2006). However, future research is crucial in order to further test these hypotheses more in-depth.

## Limitations and Future Research

This study has a number of important limitations that should be noted. First, the use of self-report instruments for the assessment of perceived parental autonomy support versus psychological control may have led to an over-estimation of the association between perceived parenting and the intervening and outcome variables. Inclusion of parent-report questionnaires is recommended for future research. Further, cross-sectional studies cannot provide evidence for whether perceived parenting indeed affects adolescents’ needs satisfaction, their developing empathic skills, their recognitions of humanness, and their tendency to engage in cyber-bullying. Longitudinal or experimental research would allow testing an alternative temporal ordering of the variables included in the model.

Further, the data were collected by a particular age period as well as in a specific cultural context, which may limit the generalizability of the findings. Cyprus is a relatively collectivisticly oriented country, where the expression of aggression and violence in interpersonal relationships may be displayed differently as compared to the individualistic ones. Similarly, it could be interesting to test whether the findings also generalize to more clinical populations.

Finally, future research would do well also to assess other relevant aspects of parenting, such as parents’ use of behavioral control (i.e., the communication of clear rules), in order to have a more holistic view and interpretation of the effects of parenting on bullying.

## Conclusion

Taken together, the findings obtained in this study shed light on the important role of the parents for understanding adolescent cyber-bullying behavior through the satisfaction of their need for autonomy, their empathic capacity towards others and their recognition of humanness to those involved in bullying behavior. Based on the above, it seems that a number of practices could be launched in order to actively support parents and schools in their role to prevent the manifestation of cyber-bullying behaviors. These practices may be taken in school environments directly, and may involve educational discussions with children regarding the effects of online bullying, or the provision of immediate information about the dehumanizing consequences of bullying or cyberbullying in school, in order to foster empathy in a non-controlling way (cf. Kaplan and Assor 2012). On the other hand, parents can respond to the theme of cyber-bullying in autonomy-supportive ways, such as discussing with children about the usage of social networking sites, forming rules for online behaviors, or proposing other media that they might enjoy (Patchin and Hinduja 2012).
